# Building consensus for the development of child eye care services in South Darfur State in Sudan using the Delphi technique

**DOI:** 10.4102/phcfm.v10i1.1767

**Published:** 2018-10-24

**Authors:** Saif H. Alrasheed, Kovin S. Naidoo, Peter C. Clarke-Farr, Kamal H. Binnawi

**Affiliations:** 1 Faculty of Optometry and Visual Science, Al Neelain University Khartoum, Sudan; 2 African Vision Research Institute, University of KwaZulu-Natal, South Africa; 3 Brien Holden Vision Institute, University of New South Wales, Australia; 4 Department of Ophthalmic Sciences, Cape Peninsula University of Technology, South Africa; 5 Faculty of Medicine, Alneelain University, Khartoum, Sudan

## Abstract

**Background:**

Global estimates suggest there are almost 19 million visually impaired children worldwide, most of whom reside in poor countries, with the major cause being treatable.

**Aim:**

To determine the barriers to accessing childhood eye care services and to develop an eye care plan for children in South Darfur State, Sudan.

**Setting:**

The study took place in South Darfur State, Sudan.

**Methods:**

The classical Delphi technique was used to build consensus on a list of statements, which were generated based on the themes established by the experts, as well as on an extensive literature review.

**Results:**

Response rates ranged from 90% in the first round (*n* = 18), 100% in the second round (*n* = 18) to 89% in the third and final round (*n* = 16). The total number of statements recommended by the Delphi panellists for development of the paediatric eye care plan, was 60 based on a consensus level of 80% agreement or more. The expert’s consensus on the following key elements for promotion and improvement of child eye care: The main barriers to accessing child eye care were high poverty rate, unavailability of child eye services and a lack of community awareness. The challenges facing visually impaired children were an absence of paediatric ophthalmologists, low vision and orthoptic services.

**Conclusion:**

The main barriers to accessing child eye care services were financial, clinical access and lack of knowledge. There should be greater collaboration between the Ministries of Health, Education and non-governmental organisations (NGOs), to work together in addressing these barriers.

## Introduction

The World Health Organization (WHO) reports that there are around 19 million visually impaired children worldwide, of whom 1.4 million are blind and 17.5 million have poor vision. Most of those children live in middle- and lower-income countries.^1,2^ In spite of visual impairment (VI) among children being less common compared to adults, it has a serious effect on the lifespan of the child.^3^ Almost 60% of children who become blind will die within one year, and those who survive have limited opportunity for education and social activities, which could increase poverty. Many causes of childhood blindness are treatable and avoidable through timely diagnosis and treatment.^4^

Sudan is the second largest country in Africa, covering an area of about 2.5 million square kilometres, with a population of more than 39 million inhabitants.^5^ The prevalence of VI and blindness among children in Khartoum (2007) was 5.5% and 2.7%, respectively.^6^ South Darfur is located in the western part of Sudan and it is the most populous state with a population of approximately 4.45 million people.^7^ The prevalence of VI among school-aged children in South Darfur State was 4.4%, where the leading causes of VI was uncorrected refractive error, affecting 57% of the children.^8^ This highlights the lack of eye care services^8^ and supports the views of Alrasheed et al.^9^ who reported that the level of knowledge and practices about eye care and refractive error was low among the students of South Darfur State in Sudan.^9^ They indicated that the fear and stigma related to the use of spectacles was widely experienced among students and their parents in Sudan, particularly among females.^10^ Sudan is a large country, with transport difficulties throughout the country as well as regional conflicts. Therefore, most hospitals, private clinics and specialists are located in the capital city, Khartoum, with approximately 70% of all eye care facilities located in this city.^9^ Most of the people in South Darfur State are of low socio-economic status and the shortage of human resources in eye care services is exacerbated by the relatively weak healthcare infrastructure. Acknowledging these challenges, the aim of this study was therefore to determine the barriers to accessing child eye care services and thereafter obtain the views of key stakeholders in order to develop a child eye care delivery plan in South Darfur State, Sudan, using the consensus-building Delphi technique.

## Methods

### Study design

The study employed the Delphi technique, which is a structured exploratory methodology that uses a series of questionnaires in order to collect information from a panel of selected experts. This process continued until consensus was reached.^11,12,13^ In this study, the classical Delphi methodology was used to develop consensus on a list of statements that had been generated through a comprehensive literature review and that included further statements obtained through feedback from the panel of experts. The group consensus level was predetermined to set consensus at 80%.

### The context of the study

Sudan has three levels of health provision: primary, secondary and tertiary. Health care services are provided by the public and private sectors in addition to the university, army, police and traditional sectors. In spite of nearly half of the population in South Darfur State being under the age of 16, there is no eye care centre for children.^6,7^ There is only an eye unit as part of the public hospitals with five eye care providers – three ophthalmologists and two optometrists – to serve a population of 4.5 million. There is an absence of paediatric eye care, orthoptics, low vision, contact lens, visual field, medical eye photography and visual function assessment services in South Darfur State. In addition, other tertiary eye hospitals provide relatively good eye services and there is a non-government hospital. The health insurance in Sudan just covers the fees of eye examinations; other eye care services such as eye drops, and spectacles are not included.^8,9^

### Participant selection

The participants in the study were representative of the three major stakeholders: service providers, policy-makers and non-governmental organisations (NGOs) working in South Darfur State, Sudan. The expert panel had equal representation from the three broad stakeholder areas. The researcher invited 20 identified experts from all sectors. The invitations and an informed consent form were sent through e-mail and a hard copy was delivered by hand. A phone call was made to ensure that the invitations were received, as well as to confirm attendance. This was done a month before the actual Delphi study. Twenty panellists from different sectors in South Darfur State consented to participate in the study.

### Inclusion criteria

The following people were included in the study:

- Members who had worked in the area for at least six years.
- Members who were suitably qualified and had a high level of knowledge about eye health care.

### Exclusion criteria

The following people were excluded from the study:

- Those who had worked in the area for less than six years.
- Those who worked in fields other than eye health care.

### Size of expert panel

There is no agreement about the optimal number of experts to comprise a panel for the Delphi method.^12^ However, several authors^11,12,13^ have indicated that the sample size and heterogeneity depends on the aim of the study, the Delphi design selected and the period of data collection. There seems to be consensus, however, that a panel of between 20 and 50 experts is most recommended.^11^ In this study, 20 panellists from different sectors in South Darfur State, Sudan, consented to participate in the study. Their selection and inclusion were based on specific criteria, and the size of the expert panel, therefore, consisted of:

- Seven eye care professionals (four optometrists and three ophthalmologists).
- Seven managers in eye health care working at primary and secondary level.
- Six members from NGOs.

### Delphi process

Initially each panellist received an information letter and consent form. The letter provided details about the study aim, the purpose of participation and information regarding withdrawal from the study. The panellists were requested to sign the consent form if they agreed to take part in the study. The invitation letters were sent to the experts to collect demographic information about the panellists such as their age, gender, employing organisation, number of years of experience, qualifications and to which professions they belonged.

### Statement development

During this phase, an open-ended questionnaire was used to seek opinions regarding barriers affecting access to eye care, as well as to collect information that could help in developing children’s eye care for South Darfur State. Each participant was asked to list as many responses (ideas, solutions, approaches, etc.) as possible. The questionnaire covered seven broad themes related to:

- The barriers to accessing childhood eye care.
- Challenges facing families with visually impaired children.
- Services and instruments needed to help children with VI.
- Important actions that should be taken to develop a children’s eye care plan.
- Suggestions for the promotion and improvement of children’s eye care services.
- Recommendations for the future regarding childhood eye care services.
- Actions that could be taken in light of the barriers to accessing children’s eye care.

After receiving feedback on this questionnaire, the collected information was assessed and grouped together under the seven overarching themes. Thereafter, the first round of Delphi statements was generated according to the themes established by the experts, as well as through an extensive literature review.^14,15,16,17,18,19,20^ The panel responses were then summarised and grouped together to establish the relevant questions and to ensure that the statements were not repetitive. Once the Delphi questionnaires were developed by researchers from the responses of experts, a pilot study was conducted where the statements were sent to other experts, who were not part of the Delphi panel. They were requested to answer the questions, check the wording and give feedback on any possible errors or any ambiguous questions and/or statements.

#### Round 1

In the first round, 20 experts who had agreed to participate in the Delphi process were sent the Delphi statement questionnaires prepared using Microsoft Excel. In addition, an instruction letter was attached on how to complete the questionnaire. The panellists were requested to rank the items according to their priorities and were also asked to rate each of the statements on the five-point Likert scale, from ‘strongly agree’ to ‘strongly disagree’. Moreover, there was space provided in front of each statement for further comments. The Delphi panellists were also requested to comment on each section, and spaces were provided in which to write comments below each overall section. The participants were asked to return the completed questionnaire within one week by e-mail or calling the principal investigator to collect the questionnaire. In this round only 18 panel members out of the 20 responded; after receiving the participants’ response, analysis was performed to determine whether the statement had reached consensus using 80% as the benchmark level.

#### Round 2

In the second round, the researcher summarised the results from the first round. Statements where consensus was reached were recorded and any statement that had not reached 80% agreement, together with any new statements generated in Round 1, were again fed back to the panellists. In this second round, each questionnaire was adapted for each participant so that they could see the response that they gave for the first round together with the percentage responses that the other panellists gave.^11,12,13^ The instruction letter was attached on how to complete the questionnaire in the second round. The panellists were instructed to reconsider if they wished to change their responses or to remain with their original choice. This version of the questionnaire was sent to the 18 Round 1 panel members who responded and all 18 (100%) completed the second round. In this round, the panellists provided further feedback and opinions on both the information and the relative importance of the items.

#### Round 3

The third round of this study was the final round, whereby the results of Round 2 were summarised in the questionnaire, showing the consensus level of each item. Any statement that had reached the consensus level of 80% agreement was excluded from the final round. The remaining statements, which had not reached consensus, as well as any new statements generated by the participants in Round 2, were sent back to the panellists, with an instruction letter on how to complete the final round. This round provided the final opportunity for the panellists to revise their judgement, and once again they were requested to rank each item according to priorities and patterns for the group consensus. They were also asked to indicate whether they agreed that they would not change their opinion again on any of the remaining items; in this case stability was considered to have been achieved. Finally, the results of all the rounds were tabulated and reported on as the final statement of group consensus.

### Analysis

Information and data obtained through the Delphi technique were captured using Microsoft Excel and the summary analysis was performed using this software. The consensus level was determined as 80% agreement. Further analysis was performed using SPSS version 22; the main statistical measures used were mean and standard deviation.

## Ethical consideration

Ethical permission for conducting the study was obtained from the University of KwaZulu-Natal’s Biomedical Research Ethics Committee (ref: BE247/14) and the National Research Ethics Review Committee in Sudan. Permission was also obtained from the South Darfur authorities in Sudan to undertake the research at their facilities. Informed consent was obtained from all the participants included in the sample study to facilitate a better understanding of conditions of involvement in the study. The research was conducted in accordance with the Declaration of Helsinki.

### Results

#### Gender and qualification of participants

The composition of the Delphi expert panel by gender and qualification is shown in Table 1. A total of 18 out of the 20 invited experts participated in the Delphi study, consisting of 14 (77.8%) males and 4 (22.2%) females. The qualifications of the expert panel were as follows: seven (38.9%) had BSc degrees, 6 (33.3%) had PhD degrees, 3 (16.7%) had diplomas and 2 (11.1) had MSc degrees.

**TABLE 1:** Gender and qualifications of participants.

Qualification	Gender of participants		Total
	Male	Female	
BSc in Medicine	1	1	2
BSc in Ophthalmic Assistance	1	0	1
BSc in Optometry	2	2	4
Diploma in Ophthalmic Assistance	2	0	2
Diploma in Optometry	1	0	1
MSc in Optometry	1	0	1
MSc in Psychology	1	0	1
PhD in Ophthalmology	2	1	2
PhD in Psychology	1	0	1
PhD in Optometry	2	0	2
**Total**	**14**	**4**	**18**
**Percent**	**77.8**	**22.2**	**100.0**

Bsc, Bachelor of Science; MSc, Master of Science; PhD, Doctor of Philosophy.

## Response rates of the Delphi panellists

In the first round, 20 panellists agreed to participate in the Delphi process but only 18 participants returned the completed questionnaires; therefore, the participants response rate was 90% for the first round. In this round most of the original statements (89%) in the Delphi questionnaire reached consensus. In addition, the expert panels suggested 12 new statements for the sections in Round 1.

In the second round, 18 of the Round 1 respondents received the Delphi questionnaire and all the panellists completed the Delphi questionnaires, giving a response rate of (100%). In this round, 88.2% of the original statements as well as the 12 new statements that were generated by the panellists in the first round reached consensus of 80% agreement or more. During this second round, the panellists recommended four new statements.

In the third and final round, 18 of the Round 2 respondents received the Delphi questionnaire but only 16 panellists completed the questionnaire, giving a response rate in the final round of 89%. Of the two panel members who did not respond in this round, one had travelled to attend a workshop and the other indicated that he was busy. In the final round, 66.7% of the statements reached consensus level. Stability was considered for those statements that did not reach consensus at this stage and were, therefore, excluded from the final barriers to eye care and the suggested childhood eye care plan. By the end of this round, there was a total of 60 statements recommended by the Delphi panellists for promotion, improvement and development of the childhood eye care plan for South Darfur State, Sudan.

## Discussion

This study utilised the Delphi technique, which aimed to determine the barriers affecting access to childhood eye care and thereafter the development of a paediatric eye care plan for South Darfur State, Sudan. Experts were asked questions regarding the barriers hindering access to children’s eye care and key components for the development of the eye care plan for children by means of assumptions, solutions and expert opinions. The statements and questions directed towards the expert panel covered seven themes related to barriers to accessing childhood eye care and the development of a paediatric eye care plan, as shown in Table 2. The Delphi process aimed to reach consensus on those aspects that should be included in the development of a child eye care plan using a group of 18 experts working in four different areas relating to eye care provision, namely ophthalmologists, optometrists, policy-makers and NGOs. The Delphi process allowed the panellists to share their views and opinions with others in a non-threatening and anonymous manner. The response rate from the panel of experts for all three rounds was high and the experts were able to generate additional statements as a result of sharing their views and opinions (Table 3). This result supports the validity of the Delphi process in this study to develop a strategy for the paediatric eye care plan. Some statements did not reach consensus during the Delphi process, but stability was reached on these statements in the final round. Each aspect of the Delphi questionnaire was discussed in turn as follows.

**TABLE 2:** Final consensus on the barriers to children’s eye care and strategies for development of children’s eye care services.

Variable	Consensus level (%)	Mean	+ S.D.	Rank
**Barriers to accessing paediatric eye care in South Darfur State**				
1. Parents are unaware of the importance of vision, childhood eye care, signs and symptoms of eye disorders.	83	3.6	± 1.04	Agree
2. The inability to pay for a child’s eye health care needs is due to the high poverty rate.	83	3.6	± 1.04	Agree
3. Many people of low-income status are unemployed and do not have insurance coverage for vision and eye health care services.	100	5.0	± 0.00	Strongly agree
4. Unavailability of eye care services in the state are a result of inequality and inappropriate distribution of eye health care providers.	89	4.7	± 1.00	Strongly agree
5. There is an absence of primary eye care services in the community and refractive error screening programmes for primary school children.	100	5.0	± 0.00	Strongly agree
6. There is a lack of paediatric eye services and insufficient training for childhood eye care providers in both the private and governmental sectors.	89	4.6	± 1.30	Strongly agree
7. There is a lack of awareness of the need for seeking preventive eye healthcare; rather eye care is sought only when there are symptoms or a problem.	89	4.6	± 1.30	Strongly agree
8. There are inadequate numbers of researchers and funding to address the diagnosis, treatment and prevalence of eye problems in South Darfur State, Sudan.	83	4.3	± 1.50	Strongly agree
9. There is a lack of regular follow-up when children have eye diseases.	89	3.7	± 1.00	Agree
10. Parents have the time to take their children to detect eye conditions and diseases, but the problem lies in the lack of health awareness.	83	3.6	± 1.04	Agree
**Challenges facing families with visually impaired children**				
1. There is a paucity of paediatric ophthalmologists and it is difficult for families to get an appointment with an eye specialist at public hospitals.	83	4.4	± 1.30	Strongly agree
2. There is a lack of low vision and orthoptics services in South Darfur State.	89	4.6	± 1.30	Strongly agree
3. There is a lack of interest in prevention and immediate treatment for eye diseases in children.	83	4.3	± 1.50	Strongly agree
4. There is a delay in the detection of children’s eye problems and difficulty committing to regular follow-up.	83	4.2	± 0.40	Agree
5. The cost of an eye examination, eyeglasses, eye drops and low vision aids is highly relevant to family income.	83	4.3	± 1.50	Strongly agree
6. There is a low level of knowledge and understanding by families of the value of eye care services.	89	4.5	± 1.10	Strongly agree
**Services and instruments needed to help children with visual impairment in South Darfur State**				
1. Establish a comprehensive vision examination protocol or programme for ophthalmologists, optometrists and other eye care personnel to conduct examinations for all children prior to entering school.	89	4.7	± 1.00	Strongly agree
2. Provide low cost or free spectacles and low vision devices to help visually impaired children at the present time.	100	5.0	± 0.00	Strongly agree
3. Supply the public hospitals with basic instruments for paediatric eye examinations such as a slit lamp, retinoscope, ophthalmoscope, orthoptics instrument and visual function assessment tools.	100	5.0	± 0.00	Strongly agree
4. Train eye care staff to deal with those children who are visually impaired in public hospitals.	100	5.0	± 0.00	Strongly agree
5. Establish rehabilitation services that enhance the vision of children with visual aids and other therapy that allow them to make possible use of their vision in education.	100	5.0	± 0.00	Strongly agree
6. Deliver health education programmes through the media to increase mothers’ and schoolchildren’s awareness of eye diseases.	100	5.0	± 0.00	Strongly agree
7. Increase health awareness for teachers in schools on how to deal with visually impaired children.	94	4.9	± 0.50	Strongly agree
8. Increase the awareness of schoolchildren on the importance of using and maintaining eyeglasses.	89	4.7	± 0.97	Strongly agree
9. The state should take care of visually impaired children in the schools and provide visual aids and mobility devices to them.	88	3.9	± 0.70	Agree
10. Train the personnel of primary health care centres and school health promotion staff and provide political and financial support for them.	81	3.6	± 0.80	Agree
Important actions that should be taken to develop a children’s eye plan for delivering eye care to children with visual impairment in South Darfur State				
1. Primary health care staff should be trained to deal with basic eye problems.	100	4.0	± 0.00	Agree
2. Staff should receive intensive training for identifying signs and symptoms and for referring eye problems during routine eye examination campaigns among schoolchildren.	100	4.0	± 0.00	Agree
3. The childhood eye plan should include the following workforce: primary eye care workers, eye care nurse, optometrists, ophthalmologists and schoolteachers.	100	5.0	± 0.00	Strongly agree
4. Referral systems and follow-up between primary, secondary and tertiary level care centres should be strengthened to ensure that services are provided at different levels.	100	5.0	± 0.00	Strongly agree
5. An eye care workforce should be trained, specifically orthoptists, low vision workers, paediatric ophthalmologists and cataract surgeons.	94	3.9	± 0.47	Agree
6. A comprehensive child eye centre should be established, with an ophthalmologist and optometrist having high skills in diagnosis and treatment of paediatric eye diseases at tertiary level.	94	4.8	± 0.70	Strongly agree
7. Key national and international partners should be engaged as a significant resource for the development and implementation of a children’s eye care plan.	94	4.8	± 0.70	Strongly agree
8. Collaboration with state and local health departments and the Ministry of Education will maximise the impact of the initiative and assure the sustainability of paediatric eye care services.	89	4.7	± 0.80	Strongly agree
9. The country should increase financial resources for the provision of eye care services and training of professionals in paediatric eye care.	100	5.0	± 0.00	Strongly agree
10. The staff of schools should be trained on health promotion to conduct eye examinations on students to reduce the risk of eye diseases.	83	4.5	± 1.20	Strongly agree
**Suggestions for the promotion and improvement of a child eye care services in South Darfur State**				
1. A national committee should be formed for improving child eye care together with all stakeholders as members.	83	3.7	± 0.84	Agree
2. The national plan for vision screening should be included as part of overall primary healthcare.	89	4.6	± 1.10	Strongly agree
3. Intensive training should be provided for primary schoolteachers to conduct eye health and vision screening of children in schools.	83	4.4	± 1.30	Strongly agree
4. Annual eye and vision screenings should be provided for students from first to eighth grade.	89	4.7	± 0.97	Strongly agree
5. The state and Ministries of Health and Education should collaborate to provide free health insurance to all schoolchildren.	100	5.0	± 0.00	Strongly agree
6. Awareness of …. eye care providers and the community should be increased on the need for childhood health services,	100	5.0	± 0.00	Strongly agree
7. Primary healthcare human resources should be strengthened to include optometrists, ophthalmic assistants and general physicians.	100	5.0	± 0.00	Strongly agree
8. There is a need to allocate special attention to eye care for the children in the camps of internally displaced people.	100	5.0	± 0.00	Strongly agree
9. The awareness of key figures in the community should be increased, for example, teachers, civil departments and religious leaders.	100	5.0	± 0.00	Strongly agree
10. Political and media support is the basis for the promotion of health services.	88	4.7	± 0.90	Strongly agree
**Recommendations for the future regarding delivery of childhood eye care services in South Darfur State**				
1. Improve and promote the existing public health infrastructure to provide childhood eye services in an effective manner including public–private partnership.	100	5.0	± 0.00	Strongly agree
2. Strengthen the partnerships and collaboration between the state government and stakeholders working in preventing avoidable childhood blindness such as non-government organisations and the private sector.	100	5.0	± 0.00	Strongly agree
3. Accelerate training initiatives of professionals for paediatric eye health such as ophthalmologists, optometrists and ophthalmic assistants to improve the quality of services.	100	5.0	± 0.00	Strongly agree
4. Provision of refractive error correction services, as well as spectacles, should be included in primary healthcare services and provided freely.	100	5.0	± 0.00	Strongly agree
5. At the level of local universities and institutions, encourage research to identify emerging childhood eye priorities and effective methods for the diagnosis and treatment of child eye diseases.	100	5.0	± 0.00	Strongly agree
6. It is very important that health insurance cover the provision of eyeglasses, eye drops and visual aids.	83	4.3	± 1.40	Strongly agree
7. There should be cooperation with other organisations and the participation of primary healthcare departments and especially curative health centres.	100	5.0	± 0.00	Strongly agree
8. There should be cooperation between eye specialists, staff assistants and gynaecology and obstetrics specialists for the care of children in the early days.	100	5.0	± 0.00	Strongly agree
9. Midwives should be trained to know the signs and symptoms of eye diseases in children because they deal more with mothers and new-borns.	88	3.8	± 0.70	Agree
**Actions that could be taken in light of the barriers to accessing child eye care in South Darfur State**				
1. Government school funds and donations by NGOs and private sectors to support the paediatric eye plan.	83	4.5	± 1.20	Strongly agree
2. Improve the quality, availability, accessibility and affordability of paediatric eye services and conduct annual vision screening for schoolchildren.	100	5.0	± 0.00	Strongly agree
3. Train community health workers, school nurses and teachers to enable early detection, referral and treatment of schoolchildren with eye disorders.	100	5.0	± 0.00	Strongly agree
4. Plan health education programmes to increase the awareness of the community on how to take care of a child’s eyes, as well as the signs, symptoms, treatment and consequences of eye disorders, through mass media (radio, television or public presentations).	100	5.0	± 0.00	Strongly agree
5. It is important to address the negative attitudes and misconceptions of parents about children’s eye problems to enhance access to existing eye care services.	89	4.7	± 0.97	Strongly agree

SD, standard deviation; NGOs, non-governmental organisations; Mean, average.

**TABLE 3:** Additional statements generated by the panellists in the first and second rounds.

Rank	± SD	Mean	Consensus level (%)	Statements generated from expert suggestions
**Agree**	± 0.65	3.8	89	There is a lack of regular follow-up when children have eye diseases.
**Agree**	± 5.50	3.8	83	Parents have the time to take their children to have their eyes checked, but the problem lies in the lack of health awareness.
**Strongly agree**	± 0.82	4.7	83	There is a lack of interest in the prevention of and immediate treatment for eye diseases in children.
**Strongly agree**	± 0.50	4.9	94	Health awareness should be increased for teachers in schools on how to deal with visually impaired children.
**Strongly agree**	± 0.97	4.7	89	The awareness of schoolchildren should be increased on the importance of using eyeglasses.
**Strongly agree**	± 1.20	4.5	83	Staff should be trained on school health promotion to conduct eye examinations for school students to reduce the risk of eye diseases.
**Strongly agree**	± 0.00	5.0	100	Primary health care should be strengthened to include optometrists, ophthalmic assistants and general physicians.
**Strongly agree**	± 0.00	5.0	100	There is a need to allocate special attention to eye care for the children in the camps of internally displaced people.
**Strongly agree**	± 0.00	5.0	100	Increase the awareness of key figures in the community, for example, teachers, civil departments and religious leaders.
**Strongly agree**	± 0.90	4.7	88	Political and media support is the basis for the promotion of health services.
**Strongly agree**	± 1.20	4.5	83	It is very important that health insurance covers eyeglasses, eye drops and visual aids.
**Strongly agree**	± 0.00	5.0	100	There should be cooperation with other organisations and the participation of primary health care departments and especially curative health centres.
**Strongly agree**	± 0.00	5.0	100	There should be cooperation between eye specialists, staff assistants and gynaecology and obstetrics specialists for the care of children in the early days.
**Agree**	± 0.70	3.8	88	Midwives should be trained to know the signs and symptoms of eye diseases in children because they deal more with mothers and new-borns.
**Agree**	± 0.70	3.9	88	The state should take care of visually impaired children in the schools and provide visual aids and mobility devices to them.
**Agree**	± 0.80	3.6	81	The personnel of primary healthcare centres and school health promotion staff should be trained, and political and financial support provided for them.

Note: Statements have been translated from the original Arabic.

SD, standard deviation; NGO, non-governmental organisations.

### Barriers to accessing paediatric eye care services

The results of the Delphi process reached broad consensus on the barriers impeding people from accessing paediatric eye care in South Darfur State (Table 2). Firstly, many people were of a low-income status, were unemployed and/or did not have health insurance coverage for vision and eye healthcare services. Further barriers included the absence of primary eye care in the community and refractive error screening programmes for primary schoolchildren. The health insurance system and the geographic distribution of healthcare providers in Sudan act as further barriers to accessing paediatric eye care and needs careful reconfiguration in the public health system, as well as the provision of comprehensive vision screening programmes for primary schoolchildren for early diagnosis and treatment of childhood eye diseases. Reports from the Sudan Federal Ministry of Health^5^ revealed that 40% of the population have no access to healthcare services. This figure masks the huge disparity between the states, where 0.1% of the population has no access in the northern part of the state compared to 42% access in West Darfur.^5^ This is comparable to that found in Western Nigeria, where the majority of eye care services are located in urban areas, leaving many rural areas without eye services.^14^ These factors are in agreement with the results of a study conducted in Ethiopia, which found that the main barriers to accessing eye services were related to the cost of services.^15^ In South Africa the results of a survey carried out by Mashige et al.^16^ showed that 80.5% of participants received the recommended treatment, yet 36.4% did not get the treatment because of affordability. A similar report by Kovai et al.^17^ found that 37% of visually impaired people in India did not seek treatment because of economic reasons.

### Challenges facing families with visually impaired children

The Delphi results showed a consensus that the difficulties facing visually impaired children were the lack of paediatric ophthalmologists, low vision care and orthoptic services. Other challenges highlighted by the Delphi panellists included delays in the detection of children’s eye problem, as well as difficulty committing to regular follow-ups (Table 2). This result agrees with a report by Borrel et al.^18^ that revealed that inequality in the distribution of eye specialists in Africa poses significant barriers to accessing eye services. For instance, 80% of ophthalmologists in Cameroon are located in 2 out of 10 provinces; in Ethiopia, most ophthalmologists are in the capital city, and in Sudan 70% of health services are based in Khartoum.^18^ About 83% of the experts strongly agreed that the barriers affecting access to paediatric eye care was the difficulty for the families in getting an appointment with eye specialists in public hospitals. The estimated population in South Darfur State is approximately 4.45 million; it is the second most populous state in Sudan.^21^ However, there is an absence of a tertiary eye hospital in the state; there is only one eye unit that is part of a public hospital, with only five eye care practitioners, consisting of three ophthalmologists and two optometrists. The Sudan Ministry of Health in 2012^22^ reported information about the distribution of health care providers, and the results show a huge disparity between the states. For example, the highest ratio of specialised doctors per population is in Khartoum State with a ratio of 54.6% per 100 000 population and the lowest is in South Darfur State, where there are 0.5% specialised doctors per 100 000 people.^22^ Almost 90% of the Delphi panel strongly agreed that the low level of knowledge and understanding of families of the value of eye care services was a major challenge for accessing child eye care. This was a result of the community’s beliefs about childhood eye diseases, many families preferring traditional medication, as well as looking at childhood eye diseases as a stage of eye development, and this may be a result of the lack of health education programmes through the public media.

### Infrastructure and human resources development

In the current study, the Delphi panellists were overwhelmingly in agreement with urgent interventions required to support child eye care and these are as follows: The lack of eye care services is the main barrier to provision of child eye care in South Darfur State, and the government and donors should help in supplying basic ophthalmic instruments such as a slit lamps, retinoscopes, ophthalmoscopes, orthoptic instruments and visual function assessment tools (Table 2). This could help low-income families access good eye care services for their children in public hospitals, as well as obtain timely diagnosis and treatment of childhood eye diseases. Most of the people in South Darfur State are below the poverty line and they cannot afford spectacles and poor vision services for their children. This in line with a study by Alrasheed et al.^9^ who reported that 80% of students from South Darfur State had never had their eyes tested and cited the cost of eye services as the biggest barrier. The government, private sector and NGOs should supply visually impaired children with the needed correction through their schools; this could help them in their education and thereafter to find employment in order to escape from poverty.

The Ministry of Health and donors need to provide financial support to eye care personnel for training in paediatric ophthalmology, as well as for the training of school health promotion staff on conducting annual vision examinations in schools, in order to detect eye problems and to refer visually impaired children. This is necessary to ensure early detection of child eye problems. Health education programmes should be established by eye care providers such as ophthalmologists and optometrists, as well as key community figures such as religious leaders, youth leaders and teachers. These programmes should be delivered through public media, television and radio in order to raise the community’s awareness of childhood eye care, the consequences of uncorrected refractive errors, as well as the serious consequences of using traditional medication in treatment of childhood eye diseases. These findings agree with those of Alrasheed et al.^10^ who revealed that fear and stigma related to the use of spectacles are widely experienced among students and their parents in South Darfur State, Sudan.

### Aspects to be addressed for the development of the paediatric eye care plan

The success of the paediatric eye care plan will depend on the following aspects, as suggested by the experts: The financial and clinical barriers are the main challenges for eye care services in developing countries. These barriers require collaborative efforts from government, NGOs and other donors. In South Darfur State, there is poor health infrastructure, a lack of paediatric eye services, as well as eye care providers who have low skills on childhood eye diseases. These challenges need urgent solutions from government as well as NGOs working on the prevention of avoidable childhood vision impairment. These aspects are comparable to those reported by the *Africa Child Policy Forum and ORBIS* (2013) about childhood eye care in Africa. The report indicated that eye health care for children in many African countries remains a major concern but is not a priority on the public health agenda.^18^ Frazier and Kleinstein^19^ indicated that the fundamentals of public health have allowed all people access to health care, and there are many public health care principles that affect availability, accessibility and affordability of eye care services. In Sudan’s public health system, primary health care does not include eye health care, and this appears to play a major role in delaying diagnosis and treatment of eye conditions. The primary care centres have no ophthalmologists, optometrists or even ophthalmic assistants. Therefore, the system of primary healthcare in Sudan needs some reconsideration to ensure that eye care services are provided through the primary health centres to cover all urban, rural and remote areas, because about two-thirds (67%) of the Sudanese population lives in rural areas.^5^ Published studies conducted in school-aged children revealed that uncorrected refractive error was a major cause of vision impairment. Therefore, a school vision screening programme would be the most effective method for detecting refractive error and other causes of vision impairment among schoolchildren. Thus, it is important to train eye care staff to identify the signs and symptoms of childhood eye conditions and refer the children with vision impairment for further diagnosis and treatment.

### The sustainability of delivering child eye services

The sustainability of delivering child eye care services is the cornerstone to implementing a successful childhood eye care plan. During the Delphi process, the panellists strongly recommended nine methods for the future regarding delivery of childhood eye care services. These recommendations included improving the existing public health infrastructure, strengthening the partnership between government and NGOs and working in preventing avoidable child blindness. Furthermore, eyeglasses and visual aids should be included in the health insurance system, and future research needs to identify emerging childhood eye health priorities (Table 2). The *Global Initiative for the Elimination of Avoidable Blindness: Action Plan 2006–2011* indicated that to eliminate the main causes of avoidable blindness, anywhere, the important thing is planning, development and implementation of sustainable eye care programmes based on three core strategies, namely disease control, human resources development and infrastructure and technology development.^20^ Moreover, Walker^4^ indicated that for building a comprehensive child vision care system in every community, public health agencies at the federal, state and local levels should facilitate the implementation of these systems in collaboration with partners from all other sectors such as health care providers, community organisations, academics, business and the media. To ensure the sustainability and effectiveness of the paediatric eye care plan in South Darfur State, multicollaboration efforts are needed from the Ministry of Health, Ministry of Education, eye care providers in the public and private sectors, NGOs, faith organisations, community leaders and local institutions.

### Actions that could be taken in light of the barriers to accessing child eye care

The barriers and factors that may affect the uptake of the eye care plan fell into categories relating to individuals’ socio-economic status, the health system and community knowledge about childhood eye care. The participants strongly agreed with the statements presented in order to overcome the barriers to accessing childhood eye care. The government, private sector and NGOs should financially support childhood eye health; there should be improvement in the existing health system – such as training of community health workers and planning of health education programmes to increase the awareness of the community about childhood eye care services (Table 2). These aspects are similar to those articulated by Jaggernath et al.^23^ in South Africa, who found that barriers that need to be considered when developing eye health interventions in poor societies include clinical barriers such as lack of skilled eye health practitioners in public eye hospitals and inadequate equipment. Moreover, there are knowledge barriers because of most people having poor knowledge about childhood eye care services. This agrees with a study by Alrasheed et al.^9^ who indicated that almost 40% of parents reported not receiving any information about child eye care. In addition, they have financial barriers and lack of funds that play a huge role in their uptake of eye care services. To eliminate the barriers affecting access to childhood eye care services in South Darfur State in Sudan, the government and other agencies should address the financial barriers by supplying public hospitals with affordable child eye care services such as spectacles and low vision services because most people in the highlighted state are below the poverty line. The public health system in this state needs some reconsideration such as having the health insurance system cover all eye care services and a school health promotion policy that includes annual vision screening. Moreover, the lack of knowledge should be eliminated by increasing childhood eye care education campaigns in order to raise community awareness through mass media, public presentations, videos in public places and leaflets handed out in public hospitals and private clinics by eye care providers.

## Limitations

There were some limitations to the Delphi aspect of the study. The Delphi panellists who participated in the current study consisted of eye care providers, policy-makers and NGOs working in South Darfur State, Sudan, resulting in a relatively small expert panel and this could have introduced some limitations to the study. During the recruitment stage of the Delphi panel, 20 experts agreed to participate in the study. However, during the Delphi process, 18 respondents participated in Rounds 1 and 2, but only 16 participants completed the final round of Delphi questionnaires.

## Conclusion

The study concluded that the cost, lack of eye care providers and lack of community awareness were the main barriers to accessing paediatric eye care in South Darfur State, Sudan. The expert panel overwhelmingly agreed that to improve paediatric eye care services in South Darfur State financial, clinical, as well as knowledge barriers should be addressed.
